# Supplementary material for: Learning curve and surgical outcome of robotic assisted colorectal surgery with ERAS program
Source: Sci Rep. 2022 Nov 29;12:20566. doi: 10.1038/s41598-022-24665-w (PMC9709162; doi:10.1038/s41598-022-24665-w)
Supplement: Supplementary file 1 — Supplementary Information 1. [file 41598_2022_24665_MOESM1_ESM.zip › LSC Raw data-Ñ[▒K/146/N_ñw╝╨░O▒Kñσ.pdf]

W52 / W52

臺中榮民總醫院
手術室護理記錄

[redacted]

基本資料

- 室別： 二醫手術室-32室，手術科別： CRS，手術類別： 常規，日期： 2021/12/16
- 進入時間： 1435 ； 麻醉開始時間： 1435 ； Time Out時間： 1550 ； 劃刀時間： 1551 ；
- 麻醉方式： GE
  診斷：Diverticulosis and diverticulitis or Ca of S colon
  手術名稱：Laparoscopic Anterior resection and anastomosis - malignant
  手術局部用藥：NIL
  術前預防性抗生素：
  - 抗生素名稱一： Veterin 1GM IVD-AR
  - 時間： 1520
  - 上次簽章: 詹佳柔
  ，追加預防性抗生素：
- 手術醫師： 蔣鋒帆，邱騰逸，林陳彥，陳怡豪，蔡濬樟
- 麻醉醫師： VS 施涵薰
- 刷手護士A ： 詹佳柔 ，時間： 1435 ，接替刷手一： 吳明柔 ，時間： 1500 ，接替刷手二： 梁凱雯 ，時間： 1900
  流動護士A ： 王姿貽 ，時間： 1435 ，接替流動一： 詹佳柔 ，時間： 1500 ，接替流動二： 陳昱穎 ，時間： 1800

皮膚狀況

A-V Shunt： 無
術前： 完整
　記錄者： 上次簽章: 詹佳柔
術後： 完整
　記錄者： 上次簽章: 陳昱穎

手術準備

手術臥式 單一手術臥式

-
  - 手術臥式： 截石臥式
  - 使用附件： 腳架
  - 皮膚準備： 10%Aq Betadine，Hibiscrub，2% Chlorhexidine
  - 電燒： 使用電燒: 電極片位置： 右大腿，記錄者： 上次簽章: 詹佳柔

止血帶T： 無

植入物

- Surgiwrap/壹/小/abdominal

, 記錄者: 上次簽章: 陳昱穎

標本送檢

- 細菌培養 無
- 細胞學 無
- 冰凍切片 無
- 病理標本
  組織名稱一：S-Colon
  ,
  組織名稱二：distal cut end
  ,
  組織名稱三：IMA root
- 截肢 無

標本交付 標本： 3 袋，標本： 0 盒，標本： 0 瓶，細胞學： 0 管，Culture Tube： 0 管，被交付醫師： Fellow 林陳彥，

- 記錄者： 上次簽章: 陳昱穎
- 時間： 1950

傷口術後數料

傷口數料： 傷口數料： 無

- 請於圖上標示部位：

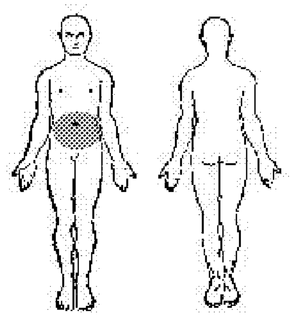

腹部 5 切口

- 傷口數料： menpore覆蓋

導管

導管	天數	導管資訊	插入日期	插入時間	更換日期	拔除日期	拔除時間	拔除原因	預計拔除/更換	預計拔除/更換日期
Foley(1)	1	號碼:16 Fr.; 部位:膀胱; 註記:BALLOON 10CC;	2021/12/16	1505						
A-line (1)	1	號碼:20 G; 部位:左手;	2021/12/16	1450						

用物計數

- 手術中出血量： minimal c.c.
- 手術中尿量： 650 c.c.
-
  - 其它： 無

用物計數： 多切口同時縫合

- 1.器械： 正確，2.縫針： 正確，3.刀片： 正確，4.紗布： 正確，5.腹部墊： 未使用，6.紗球： 未使用，7.PEANUTS ： 未使用，8.紗條： 未使用，9.絲帶： 未使用，10.針頭： 未使用，11.Vessel loop： 未使用，12.Boots： 未使用，13.Jelco： 未使用，14.棉條： 未使用，
  - 第一次：
    - 刷手護士 ： 梁凱雯
    - 流動護士： 上次簽章: 陳昱穎
    - 時間： 1900

  ,
  - 第二次：
    - 刷手護士 ： 梁凱雯
    - 流動護士： 上次簽章: 陳昱穎
    - 時間： 1920

  ,
  - 第三次：
    - 刷手護士 ： 梁凱雯
    - 流動護士： 上次簽章: 陳昱穎
    - 時間： 1950

備註

備註：NIL

- 記錄者： 上次簽章: 陳昱穎
- 時間： 1800

填寫護理人員 陳昱穎

正式摘要

	W52 / W52

doctorRank:B
